# Evaluating the associations between intelligence quotient and multi-tissue proteome from the brain, CSF and plasma

**DOI:** 10.1093/braincomms/fcae207

**Published:** 2024-06-26

**Authors:** Sirong Shi, Yujing Chen, Xiaoge Chu, Panxing Shi, Bingyi Wang, Qingqing Cai, Dan He, Na Zhang, Xiaoyue Qin, Wenming Wei, Yijing Zhao, Yumeng Jia, Feng Zhang, Yan Wen

**Affiliations:** NHC Key Laboratory of Environment and Endemic Diseases, School of Public Health, Health Science Center, Xi’an Jiaotong University, Xi’an, Shaanxi, 710061, China; NHC Key Laboratory of Environment and Endemic Diseases, School of Public Health, Health Science Center, Xi’an Jiaotong University, Xi’an, Shaanxi, 710061, China; NHC Key Laboratory of Environment and Endemic Diseases, School of Public Health, Health Science Center, Xi’an Jiaotong University, Xi’an, Shaanxi, 710061, China; NHC Key Laboratory of Environment and Endemic Diseases, School of Public Health, Health Science Center, Xi’an Jiaotong University, Xi’an, Shaanxi, 710061, China; NHC Key Laboratory of Environment and Endemic Diseases, School of Public Health, Health Science Center, Xi’an Jiaotong University, Xi’an, Shaanxi, 710061, China; NHC Key Laboratory of Environment and Endemic Diseases, School of Public Health, Health Science Center, Xi’an Jiaotong University, Xi’an, Shaanxi, 710061, China; NHC Key Laboratory of Environment and Endemic Diseases, School of Public Health, Health Science Center, Xi’an Jiaotong University, Xi’an, Shaanxi, 710061, China; NHC Key Laboratory of Environment and Endemic Diseases, School of Public Health, Health Science Center, Xi’an Jiaotong University, Xi’an, Shaanxi, 710061, China; NHC Key Laboratory of Environment and Endemic Diseases, School of Public Health, Health Science Center, Xi’an Jiaotong University, Xi’an, Shaanxi, 710061, China; NHC Key Laboratory of Environment and Endemic Diseases, School of Public Health, Health Science Center, Xi’an Jiaotong University, Xi’an, Shaanxi, 710061, China; NHC Key Laboratory of Environment and Endemic Diseases, School of Public Health, Health Science Center, Xi’an Jiaotong University, Xi’an, Shaanxi, 710061, China; NHC Key Laboratory of Environment and Endemic Diseases, School of Public Health, Health Science Center, Xi’an Jiaotong University, Xi’an, Shaanxi, 710061, China; NHC Key Laboratory of Environment and Endemic Diseases, School of Public Health, Health Science Center, Xi’an Jiaotong University, Xi’an, Shaanxi, 710061, China; NHC Key Laboratory of Environment and Endemic Diseases, School of Public Health, Health Science Center, Xi’an Jiaotong University, Xi’an, Shaanxi, 710061, China

**Keywords:** tissue protein, MSP, IQ trait, brain disease

## Abstract

Intelligence quotient is a vital index to evaluate the ability of an individual to think rationally, learn from experience and deal with the environment effectively. However, limited efforts have been paid to explore the potential associations of intelligence quotient traits with the tissue proteins from the brain, CSF and plasma. The information of protein quantitative trait loci was collected from a recently released genome-wide association study conducted on quantification data of proteins from the tissues including the brain, CSF and plasma. Using the individual-level genotypic data from the UK Biobank cohort, we calculated the polygenic risk scores for each protein based on the protein quantitative trait locus data sets above. Then, Pearson correlation analysis was applied to evaluate the relationships between intelligence quotient traits (including 120 330 subjects for ‘fluid intelligence score’ and 38 949 subjects for ‘maximum digits remembered correctly’) and polygenic risk scores of each protein in the brain (17 protein polygenic risk scores), CSF (116 protein polygenic risk scores) and plasma (59 protein polygenic risk scores). The Bonferroni corrected *P*-value threshold was *P* < 1.30 × 10^−4^ (0.05/384). Finally, Mendelian randomization analysis was conducted to test the causal relationships between ‘fluid intelligence score’ and pre-specific proteins from correlation analysis results. Pearson correlation analysis identified significant association signals between the protein of macrophage-stimulating protein and fluid intelligence in brain and CSF tissues (*P*_brain_ = 1.21 × 10^−8^, *P*_CSF_ = 1.10 × 10^−7^), as well as between B-cell lymphoma 6 protein and fluid intelligence in CSF (*P*_CSF_ = 1.23 × 10^−4^). Other proteins showed close-to-significant associations with the trait of ‘fluid intelligence score’, such as plasma protease C1 inhibitor (*P*_CSF_ = 4.19 × 10^−4^, *P*_plasma_ = 6.97 × 10^−4^), and with the trait of ‘maximum digits remembered correctly’, such as tenascin (*P*_plasma_ = 3.42 × 10^−4^). Additionally, Mendelian randomization analysis results suggested that macrophage-stimulating protein (Mendelian randomization-Egger: *β* = 0.54, *P* = 1.64 × 10^−61^ in the brain; *β* = 0.09, *P* = 1.60 × 10^−12^ in CSF) had causal effects on fluid intelligence score. We observed functional relevance of specific tissue proteins to intelligence quotient and identified several candidate proteins, such as macrophage-stimulating protein. This study provided a novel insight to the relationship between tissue proteins and intelligence quotient traits.

## Teaser

Shi *et al*. report the functional relevance of certain tissue proteins to intelligence quotient and identified several candidate proteins, such as macrophage-stimulating protein. Mendelian randomization analysis suggested macrophage-stimulating protein showed causal effects on fluid intelligence score. This study provided a novel insight to further understand the mechanism of intelligence quotient level.

## Graphical Abstract

**Graphical Abstract:**
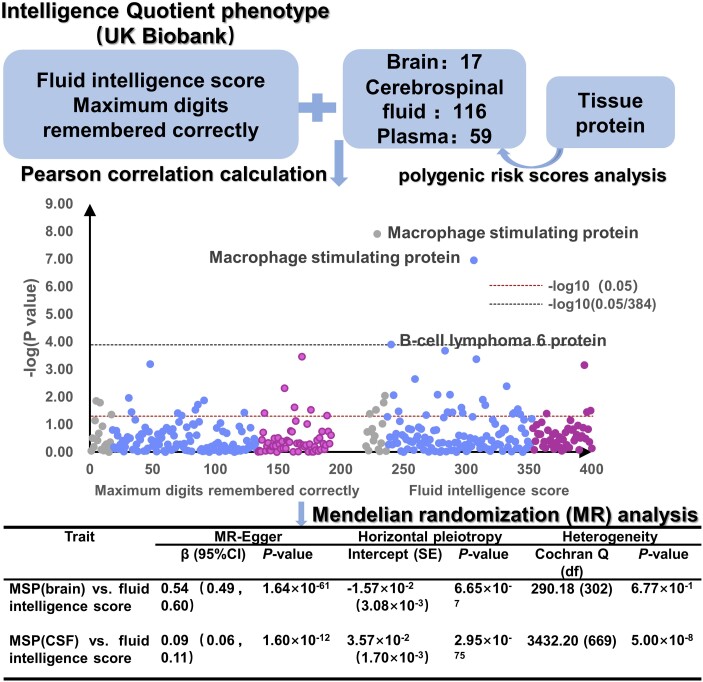

## Introduction

Intelligence is referred to as the ability of an individual to think rationally, learn from experience and deal with the environment effectively.^1^ Intelligence quotient (IQ) is expressed as a score calculated from a standardized test and can be used as an indicator of an individual’s intelligence level.^2^ IQ is important for predicting an individual performance in education, self-care, social well-being and employment in later life.^3^ Previous study revealed that the IQ score had a foundation in the structure and function of brain.^4^ People with higher IQ scores have faster nerve conduction velocity and lower brain metabolic rates under a mentally active state.^5^ In this study, the phenotype of IQ is measured by two individual scores, including ‘fluid intelligence score’ and ‘maximum digits remembered correctly’. Fluid intelligence indicates the ability to think and reason abstractly and solve a new problem that generally cannot be relied on existing experience.^6^ ‘Maximum digits remembered correctly’ is a measure of one’s ability to maintain items in memory for short periods of time and manipulate them.^7^

IQ value, which can predict education, occupational and health outcomes, has high heritability.^8^ Meta-analysis suggests an overall heritability of IQ around 0.54.^9^ A substantial proportion of individual differences in human intelligence is thought to result from genetic variation.^10^ A correlation study has found that the maternal IQ and the children’s IQ were highly related.^11^ Further, environmental factors can also affect changes in IQ scores.^12^ A study has found that various environmental factors such as place of residence, physical exercise and malnutrition can influence the IQ of a child to a great extent.^13^ Besides, the education, occupation and income of parents—indices of the families’ socioeconomic status—have been found to moderate their children’s IQ.^14^

Proteins are defined as the end products of gene expression and the main functional component of cell and biological processes.^15^ Proteome composition of the brain, CSF and plasma can reflect the brain structure and function.^16^ Previous studies found that some specific proteins are related to IQ or neurodegenerative diseases, such as NRX1A, LTBP4, periostin and CD70.^17^ For example, abnormally phosphorylated tau protein can affect synaptic events that are critical to memory formation. It is also a key marker of Down syndrome and frontotemporal dementia.^18^ Disease-related proteins were identified to exist in the brain and CSF of Alzheimer’s disease patients.^18^ 14-3-3γ protein is thought to play an important role in neural development.^19^ It can interact with tau proteins and stimulate tau phosphorylation, which might be the underlying mechanism in neurodegeneration.^20^ Some studies also have revealed that the abnormal proteinaceous, such as Lewy bodies and Lewy neurites, form intracerebrally during the progression of synucleinopathies, a kind of idiopathic Parkinson’s disease.^21^ However, the exact specific kinds of proteins related to IQ and the underlying biological foundation of IQ feature remain largely unknown until now.

In summary, we hypothesized that the IQ levels could be affected by the relevant proteins in multiple tissues such as the brain, CSF and plasma, whose levels can be predicted and estimated by genetic components. Firstly, we calculated polygenic risk score (PRS) for each protein in three tissues (brain, CSF and plasma) in the UK Biobank population based on a reference SNPs (single nucleotide polymorphisms) list derived from previous study. Then, Pearson correlation analysis was conducted to evaluate the associations between IQ traits and the protein PRSs. Further, Mendelian randomization (MR) analysis was conducted to test the causal relationship between ‘fluid intelligence score’ and the protein of macrophage-stimulating protein (MSP) and B-cell lymphoma 6 protein (BCL6). MSP showed significant correlation in the above analysis.

## Materials and methods

### UK Biobank data set

The analysis data of study individuals were extracted from the UK Biobank health resource (http://www.ukbiobank.ac.uk/about-biobank-uk/). The UK Biobank study is a large prospective cohort study involving 502 656 individuals aged from 37 to 76 years (99.5% were aged 40–69 years) whose residences are within the UK. All participants provided a range of information on health status, demographics and lifestyle via questionnaires and interviews.^22^ The genotypes of the UK Biobank participants were assayed using either the Affymetrix UK BiLEVE Axiom or Affymetrix UK Biobank Axiom array. Imputation was conducted by IMPUTE4 against the reference panel of the Haplotype Reference Consortium, 1000 Genomes and UK10K projects. We used the genetic data with imputed SNP information in it that is released by the UK Biobank in July 2017.^23^ Full details regarding these data have been published elsewhere.^24^ The UK Biobank has ethical approval from the Northwest Multicentre Research Ethics Committee, and informed consent was obtained from all participants. This research was conducted using the UK Biobank resource under application number 46478.

### The phenotypic definition of IQ score

Then, we included the subjects with the phenotype of ‘fluid intelligence score’ (data field: 20016) and ‘maximum digits remembered correctly’ (data field: 4282) as IQ traits in this study from the UK Biobank (https://biobank.ctsu.ox.ac.uk/showcase/search.cgi). As defined in the UK Biobank, ‘fluid intelligence’ describes ‘the capacity to solve problems that require logic and reasoning ability, independent of acquired knowledge’. The questionnaire was conducted on touch screen in the assessment centre of the UK Biobank. It included a total of 13 questions such as ‘Which number is the largest? (options included a series of number as well as do not know and prefer not to answer)’, ‘Age is to years as height is to? (options included long, deep, top, metres, tall and do not know and prefer not to answer)’ and ‘Divide the sixth number to the right of twelve by three. Is the answer? (options included a series of number and do not know and prefer not to answer)’. The sum was then calculated based on the individual score of each question. More detailed information can be seen at https://biobank.ctsu.ox.ac.uk/showcase/refer.cgi?id=100231. For ‘maximum digits remembered correctly’, the UK Biobank defines it as ‘the longest number that can be recalled correctly during the numeric memory test’. In this test, the participant was firstly given a two-digit number to remember on the touch screen. After a short time, the number disappeared, and then, they were asked to type the exact number just in the order they have seen. The number became 1 digit longer each time after they remembered the previous one correctly (up to a maximum of 12 digits). A value of −1 is recorded if the participant chose to quit the test before completing the first round. More detailed information about this test can be seen at https://biobank.ctsu.ox.ac.uk/showcase/refer.cgi?id=6. The exclusion criteria included the subjects (i) without the information of covariates (seen in Pearson correlation analysis section below) including sex, age, 10 principal components of population structure, smoking, alcohol use and Townsend deprivation index; (ii) who did not finish the questions in the required time for ‘fluid intelligence score’ test; (iii) who chose to abandon the test before completing the first round for ‘maximum digits remembered correctly’ test; and (iv) who had a self-reported gender inconsistent with the genetic gender or who withdraw their consents.

### PRS data sets of tissue proteome

The PRS of tissue proteins was calculated on the basis of a previous study results, which released a genomic atlas of proteomes in the brain, CSF and plasma.^16^ The data sets of 17 proteins in the brain, 116 proteins in CSF and 59 proteins in plasma were collected from this study (https://www.ncbi.nlm.nih.gov/pmc/articles/PMC8521603/).^16^ In this study, proteins of 3 tissues were extracted from 1537 participants. Whole-proteomic quantification were analysed on multiplexed, aptamer-based platform.^16^ A total of 14 059 245 genotyped and imputed SNPs were used for subsequent analysis. To identify the protein quantitative trait loci (pQTLs) that can regulate the protein level, a linear regression was performed under additive model with age, sex, principal component factors from population stratification and genotype platform as covariates.^16^ We extracted the summary data sets of the results. Detailed sample collection, proteomic and genomic data QC process, pQTL identification and annotation of pQTL can be seen in the original study.^16^

### PRS analysis

Before we calculated the PRSs for proteins, we further filter the SNPs (or pQTLs) obtained from original data sets as mentioned above^16^ to form our PRS reference SNP list. The exclusion criteria applied for the whole data set of original SNP list included (i) the SNPs with *r*^2^ ≥ 0.2 (remained the one with smaller *P*-value), (ii) the SNPs not available in the genotype of the UK Biobank data set and (iii) the proteins that only have one associated SNP. Finally, we included 57 SNPs in brain tissue, 793 SNPs in CSF tissue and 271 SNPs in plasma tissue for PRS calculation. The PRS of each protein was computed in PLINK 2.0 (http://www.cog-genomics.org/plink/2.0/) based on the individual-level genotype data of the UK Biobank according to the classic approach.^25^ The methods are as follows. $\mathrm{PR}S_{m}$ denotes the PRS value of pQTL for the *m*th subject, and it is defined as


$$\mathrm{PR}S_{m}=\sum_{i=1}^{l}\beta_{i}\mathrm{SN}P_{im},$$

where $\beta_{i}$ is the effect parameter of risk allele of the *i*th significant pQTL, $\mathrm{SN}P_{im}$ is the dosage (0, 1 and 2) of the risk allele of the ith SNPs for the mth study subjects and *l* denotes the total number of pQTL-associated SNPs. Finally, we obtained a data set of protein PRS in 3 tissues, which specifically included 17 protein PRSs in the brain, 116 protein PRSs in CSF and 59 protein PRSs in plasma (Supplementary Table 1).

### Pearson correlation analysis

We performed Pearson correlation analysis with IQ traits as dependent variant and protein PRSs as independent variant by the function ‘lm’ of R 3.5.3. First, we used linear model to adjust the IQ scores with sex, age, 10 principal components of population structure, smoking, alcohol use and Townsend deprivation index (Supplementary material—Covariate data) by the function ‘lm’ of R 3.5.3, with the formula as the following:


$$\begin{array}{rl} & \mathrm{lm}\;(\mathrm{IQ}\;\mathrm{simage}+\mathrm{sex}+\mathrm{PC}1+\mathrm{PC}2+\mathrm{PC}3+\mathrm{PC}4+\mathrm{PC}5\\ & \;+\mathrm{PC}6+\mathrm{PC}7+\mathrm{PC}8+\mathrm{PC}9+\mathrm{PC}10+\mathrm{alcohol}\;\mathrm{use}\\ & \;+\mathrm{smoking}+\mathrm{Townsend}\;\mathrm{index}). \end{array}$$

Then, Pearson correlation analysis was conducted to evaluate the association between IQ score and proteins by using the residuals regressed out above and the PRSs of the proteins. In summary, we conducted the correlation analysis between 192 protein PRSs (17 in the brain, 116 in CSF and 59 in plasma; Supplementary Table 1) and two IQ traits (‘fluid intelligence score’ and ‘maximum digits remembered correctly’). After Bonferroni multiple testing correction, the significance threshold was at *P* < 1.30 × 10^−4^ (0.05/384, analysis in 192 protein PRSs for 2 phenotypes). All analyses were conducted by R 3.5.3.

### MR analysis of MSP and IQ trait

The causal relationship between the identified proteins in regression analysis (MSP and BCL6) and ‘fluid intelligence score’ from the UK Biobank was assessed by MR method, under different assumptions including inverse variance-weighted, MR-Egger regression, MR-Pleiotropy RESidual Sum, weighted-median, simple mode and weighted mode methods. We used the Cochran *Q* test to detect heterogeneity.^26^ Horizontal pleiotropy occurs when a genetic variant affects the outcome variable through pathways other than or in addition to the exposure variable.^27^ When horizontal pleiotropy is unbalanced, which means genetic variants are associated with the outcome through pathways other than the exposure, the MR-Egger method provides an unbiased effect estimate that could balance horizontal pleiotropy.^28^ Therefore, in the presence of pleiotropy, an MR-Egger regression estimate was used. Briefly, we incorporated the significant SNPs with *P* < 5 × 10^−8^. We finally included a total of 304 MSP-associated SNPs in the brain and a total of 671 MSP-associated SNPs in CSF for MR analysis. The MR analysis was performed using the MR package for R 3.5.3.^29^

### Institutional review board statement

This study has been approved by UKB (application 46478) and obtained participants’ health-related records.

### Informed consent statement

Informed consent was obtained from all subjects involved in UKB.

## Results

### Baseline characteristics of IQ traits

For assessing the correlation of ‘fluid intelligence score’ and protein PRS, we eventually incorporated a population of 120 330 subjects from the UK Biobank cohort, with an average age of 57.30 ± 7.94, which included 64 342 females and 55 988 males. The average value of ‘fluid intelligence score’ was at 6.18 ± 2.10. For ‘fluid intelligence score’, a total of 221 subjects (a value of 0) who did not answer all of the questions within the allotted 2 min limit are scored as 0 for each of the questions. And for ‘maximum digits remembered correctly’ and protein PRS correlation analysis, we incorporated a population of 38 949 subjects from the UK Biobank cohort, with an average age of 56.93 ± 8.10, which included 20 965 females and 17 984 males. The mean value of ‘maximum digits remembered correctly’ was at 6.71 ± 1.32. For ‘maximum digits remembered correctly’, a total of 1444 subjects (a value of −1), who chose to abandon the test before completing the first round, were then excluded.

### Correlations between IQ score and tissue protein PRS

We further conducted correlation between 192 protein PRSs and IQ traits. For ‘fluid intelligence score’ feature, we identified significant correlation signals at the protein of MSP in the brain (*P* = 1.21 × 10^−8^) and CSF (*P* = 1.10 × 10^−7^), as well as BCL6 in CSF (*P* = 1.23 × 10^−4^). There were also some other tissue proteins that showed close-to-significant signal with fluid intelligence score, such as apolipoprotein E (isoform E2, APOE; *P* = 1.59 × 10^−2^ in the brain), plasma protease C1 inhibitor (C1-INH; *P* = 4.19 × 10^−4^ in CSF; *P* = 6.97 × 10^−4^ in plasma) and granzyme A (GZMA; *P* = 2.08 × 10^−4^ in CSF).

**Figure 1:**
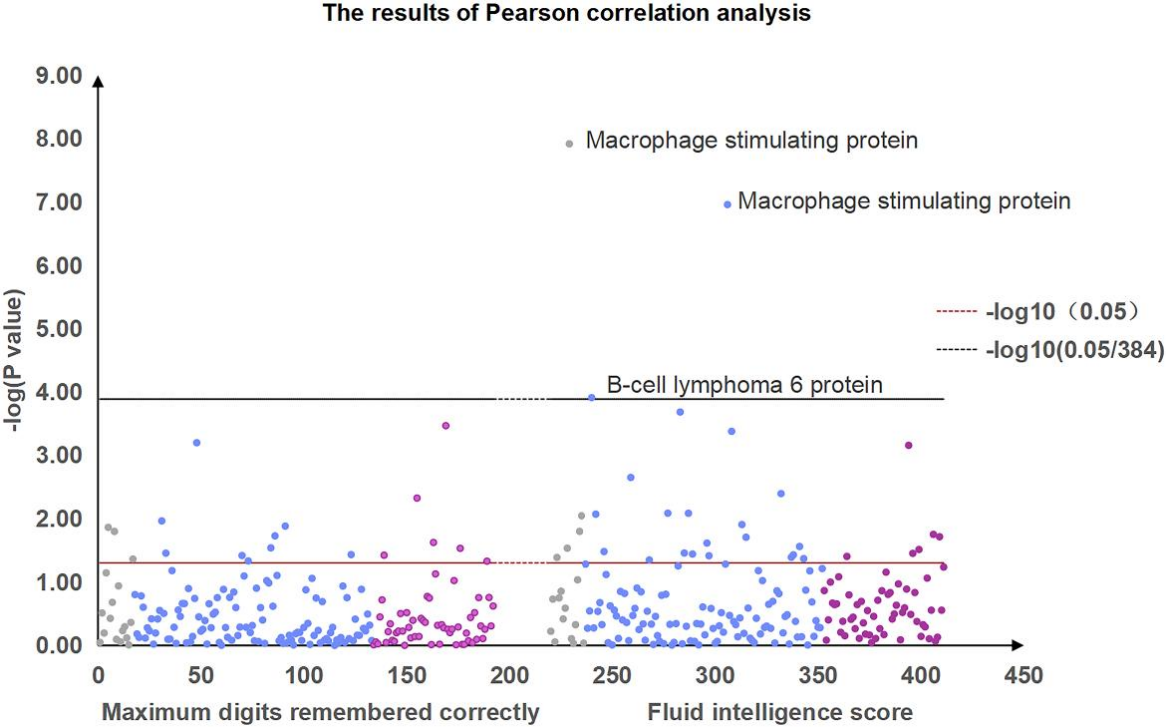
**The results of Pearson correlation analysis.** The scatter plot of the results obtained by −log10 *P*-value of the association between imputed protein level (brain, CSF and plasma) and IQ traits. The order of *x*-axis is brain (grey), CSF (blue) and plasma (purple) for maximum digits remembered correctly and is brain (grey), CSF (blue) and plasma (purple) for fluid intelligence score. *y*-axis represents the negative logarithm of *P*-values (from Pearson regression analysis). The horizontal black line in the figure represents −log10 (0.05). The horizontal red line in the figure represents −log10 (0.05). The Bonferroni correction *P* < −log10 [1.30 × 10^−4^ (0.05/384)] was considered significant, and *P* < 0.05 was considered close to significant. MSP, macrophage-stimulating protein; BCL6, B-cell lymphoma 6 protein; CSF, cerebrospinal fluid.

For ‘maximum digits remembered correctly’ feature, we did not identify significant correlation signals. The top smallest *P*-values were seen at the protein of N-acylethanolamine-hydrolyzing acid amidase (ASAHL; *P* = 1.37 × 10^−2^ in the brain; *P* = 6.35 × 10^−4^ in CSF; *P* = 4.74 × 10^−3^ in plasma) and tenascin (TNC; *P* = 3.42 × 10^−4^ in plasma; Table 1 and Figure 1).

**Table 1:** The result of Pearson correlation analysis between tissue proteins and IQ traits

IQ trait	Protein	Tissue	*r*	*P*-value
Fluid intelligence score	MSP	Brain	1.64 × 10^−2^	1.21 × 10^−8^
	APOE	Brain	6.95 × 10^−3^	1.59 × 10^−2^
	CPNE1	Brain	−7.53 × 10^−3^	9.00 × 10^−3^
	MSP	CSF	1.53 × 10^−2^	1.10 × 10^−7^
	BCL6	CSF	1.11 × 10^−2^	1.23 × 10^−4^
	GZMA	CSF	−1.07 × 10^−2^	2.08 × 10^−4^
	IL1R1	CSF	8.81 × 10^−3^	2.24 × 10^−3^
	C1-INH	CSF	1.02 × 10^−2^	4.19 × 10^−4^
	C1-INH	Plasma	9.78 × 10^−3^	6.97 × 10^−4^
	SEMA3E	Plasma	6.84 × 10^−3^	1.77 × 10^−2^
Maximum digits remembered correctly	AKR1A1	Brain	1.22 × 10^−2^	1.59 × 10^−2^
	ASAHL	Brain	1.25 × 10^−2^	1.37 × 10^−2^
	ASAHL	CSF	1.73 × 10^−2^	6.35 × 10^−4^
	IL-22	CSF	1.29 × 10^−2^	1.09 × 10^−2^
	CDON	CSF	1.26 × 10^−2^	1.31 × 10^−2^
	ICAM-1	CSF	1.19 × 10^−2^	1.87 × 10^−2^
	ASAHL	Plasma	1.43 × 10^−2^	4.74 × 10^−3^
	TNC	Plasma	1.81 × 10^−2^	3.42 × 10^−4^
	CAMK1	Plasma	1.15 × 10^−2^	2.38 × 10^−2^
	BST1	Plasma	−1.10 × 10^−2^	2.94 × 10^−2^

The Bonferroni correction *P* < 1.30 × 10^−4^ (0.05/384) was considered significant, and *P* < 0.05 was considered suggestively significant. MSP, macrophage-stimulating protein; APOE, apolipoprotein E (isoform E2); CPNE1, Copine-1; BCL6, B-cell lymphoma 6 protein; GZMA, granzyme A; IL1R1, interleukin-1 receptor type 1; C1-INH, plasma protease C1 inhibitor; SEMA3E, semaphorin-3E; AKR1A1, alcohol dehydrogenase [NADP(+)]; IL-22, interleukin-22; CDON, cell adhesion molecule-related/downregulated by oncogenes; ICAM-1, intercellular adhesion molecule 1; ASAHL, N-acylethanolamine-hydrolyzing acid amidase; TNC, tenascin; CAMK1, calcium/calmodulin-dependent protein kinase type 1; BST1, ADP-ribosyl cyclase/cyclic ADP-ribose hydrolase 2; SE, standard error.

### MR analysis

In this study, we focused on MR-Egger model for MR analysis and detected a positively causal relationship between MSP (exposure) and the fluid intelligence score [outcome; brain: *β*, 0.54; 95% confidence interval (CI), 0.49, 0.60; *P* = 1.64 × 10^−61^; CSF: *β*, 0.09; 95% CI, 0.06, 0.11; *P* = 1.60 × 10^−12^]. However, we did not find the causal relationship between BCL6 (exposure) and the ‘fluid intelligence score’ (outcome; Table 2; Supplementary Table 2).

**Table 2:** The result of MR analysis of MSP (exposure) and fluid intelligence score (outcome)

Trait	MR-Egger		Horizontal pleiotropy		Heterogeneity	
	*β* (95% CI)	*P*-value	Intercept (SE)	*P*-value	Cochran *Q* statistics (df)	*P*-value
MSP (brain) versus fluid intelligence score	0.54 (0.49, 0.60)	1.64 × 10^−61^	−1.57 × 10^−2^ (3.08 × 10^−3^)	6.65 × 10^−7^	290.18 (302)	6.77 × 10^−1^
MSP (CSF) versus fluid intelligence score	0.09 (0.06, 0.11)	1.60 × 10^−12^	3.57 × 10^−2^ (1.70 × 10^−3^)	2.95 × 10^−75^	3432.20 (669)	5.00 × 10^−8^

Estimates of the *β* (95% CI) *P*-value from MR-Egger, estimates of the Cochran *Q* statistics (df) *P*-value from Cochran *Q* test and estimates of the intercept (SE) *P*-value from MR-Egger-intercept. *P* < 0.05/2 indicates statistical significance. MR, Mendelian randomization; MSP, macrophage-stimulating protein; IQ, intelligence quotient; CSF, cerebrospinal fluid; SE, standard error; df, degree of freedom.

## Discussion

Although previous studies have found the functional relevance of tissue proteins and the development of brain function, the underlying biological mechanism between tissue proteins and IQ traits remains to be elucidated.^30^ In this study, the associations between tissue proteins—especially MSP and ‘fluid intelligence score’—were detected by Pearson correlation analysis. MR analysis also found the positively causal relationship between MSP and ‘fluid intelligence score’.

In this study, we identified that MSP is the main linked protein to IQ traits that showed strong correlation signal and a potential causal effect. MSP, also known as hepatocyte growth factor–like protein, is the only protein identified as a specific ligand for tyrosine kinase receptor (RON), a protein expressed by microglia in brain.^31^ In addition to stimulating macrophages, MSP can also act with other cell types including epithelial and haematopoietic cells.^32^ After intracerebral haemorrhage (ICH), MSP and RON are found to be expressed less in neurons and predominantly in astrocytes, which is a kind of macrophage and closely related to the neural degeneration, regeneration and cerebral ischaemic injury.^33,34^

MSP takes part in multiple neurological disorders. MSP–RON cascade can regulate neuroinflammation process by exerting neuroprotective effect in multiple sclerosis and experimental autoimmune encephalomyelitis.^35^ MSP-deficient animals with experimental autoimmune encephalomyelitis induction showed an earlier onset of neurological dysfunctions such as exacerbated demyelination, axonal injury and neuroinflammation.^35^ Another study revealed that MSP is over-expressed and activated in malignant gliomas, which can stimulate glioma proliferation, migration, invasion and vascularization *in vivo*.^36^ MSP is also identified to be able to preserve blood brain barrier integrity and reduce brain oedema and neurological deficits after ICH.^33^ Zhou *et al*.^37^ found that MSP upregulation occurs after cerebral ischaemia reperfusion (IR) injury, while the genetic ablation of MSP can attenuate mitochondrial damage and sustain brain function following cerebral IR injury. Additionally, MSP can inhibit brain metastases of melanoma through suppression of mitogen-activated protein kinase (MAPK) pathway activation.^38^

MSP is present in human brain perivascular macrophages and microglia,^35^ which play critical roles in initiating and sustaining immune responses to microbial, neoplastic and neural antigens in the CNS.^39^ The activation of microglia and secretion of pro-inflammatory molecules play critical roles in the pathogenesis of CNS inflammatory diseases including multiple sclerosis, Alzheimer’s disease^40^ and HIV-associated dementia.^41^ Alzheimer’s disease is the most common cause of progressive intellectual failure.^42^ MSP is also a novel neurotrophic factor for cranial motoneurons and regeneration by regulating the production of nitric oxide.^43^ IQ and brain function would be affected through these mechanisms in varying degrees. Although there are a body of evidence that proved the important role of MSP in neural system, limited efforts are paid to elucidate its implication in IQ trait. MSP-linked molecular mechanism underlying IQ traits should a novel direction for future studies of this protein.

We also identified a set of close-to-significant tissue proteins associated with IQ traits, such as TNC, GZMA, C1-INH and APOE.

TNC is an extracellular matrix glycoprotein that is expressed during embryonic CNS development.^44^ It is also a key modulator of the immune response during neurodegeneration in the CNS.^44^ In adults, TNC is present at low levels. However, it increases under pathological conditions such as in brain tumours, injury and neurodegenerative disorders.^44^ TNC acts as a mediator of neuronal apoptosis via activation of platelet-derived growth factor receptor and MAPK in subarachnoid haemorrhage–induced brain injuries.^45^ Besides, TNC is involved in the development of Alzheimer’s disease and human gliomas.^46^ GZMA, a serine protease mainly expressed by natural killer and T cells, could act as a pro-inflammatory mediator and play an important role in the pathogenesis of sepsis.^47^ Recent study indicated the involvement of GZMA in the pathogenesis of acute encephalopathy.^48^ It is reported that BCL6 exhibited aberrant levels in neurogenesis and neurological diseases.^49^ Knockdown of BCL6 partially rescued neuronal death, while BCL6 over-expression increased neuronal death.^50^ C1-INH, also known as complement component 1 inhibitor, is expressed in the blood and brain.^51^ Parkinson’s disease occurs after the death of dopaminergic neurons in the substantia nigra.^52^ A study has showed that a decrease in C1-INH inhibited dopaminergic cell reduction and consequently a reduction in Parkinson’s disease pathology.^53^

To the best of our knowledge, this is a study to systematically explore the relationship between tissue proteins and IQ traits based on large genome-wide association study (GWAS) data sources. Our study provides novel clues for the mechanism study of IQ at the protein level. However, there are still some limitations that should be noted. Firstly, the tissue proteome from the brain, CSF and plasma associated with SNP sets was obtained from previous GWAS. The accuracy of our correlation analysis may be affected by the power of previous GWAS of the tissue proteins. Secondly, because we only analysed the genetic data from European cohorts in this study, we should be careful to apply our results to other populations. Thirdly, although the MR-Egger method’s estimate is known to be relatively robust to the presence of pleiotropy, it is also affected by a reduced statistical power. Further studies with other independent samples and biological studies are needed to confirm our findings.

In conclusion, our results support the significant associations between tissue proteins and IQ traits and identified a few of potentially linked proteins for IQ traits. These findings could provide novel insights into the biological studies of IQ and neurological disease. Further studies are warranted to confirm our findings and reveal the biological mechanism underlying the observed association.

## Supplementary Material

fcae207_Supplementary_Data

## Data Availability

The UK Biobank data are available through the UK Biobank Access Management System (https://www.ukbiobank.ac.uk/). We will return the derived data fields following the UK Biobank policy; in due course, they will be available through the UK Biobank Access Management System. The Comparative Toxicogenomics Database is available through the Comparative Toxicogenomics Database Access Management System (https://ctdbase.org/). We will return the derived data fields following CTD policy; in due course, they will be available through the CTD Access Management System.
