# Supplementary material for: Evaluating the associations between intelligence quotient and multi-tissue proteome from the brain, CSF and plasma
Source: Brain Commun. 2024 Jun 26;6(4):fcae207. doi: 10.1093/braincomms/fcae207 (PMC11220507; doi:10.1093/braincomms/fcae207)
Supplement: fcae207_Supplementary_Data [file fcae207_supplementary_data.pdf]

Covariate data

The following covariate data were also utilized:

sex

We selected the sex in the UKB (Data-Field 31). Acquired from central registry at recruitment, but in some cases updated by the participant. Hence this field may contain a mixture of the sex the NHS had recorded for the participant and self-reported sex.

age

We selected the age in the UKB (Data-Field 21022). This is a derived variable based on date of birth and date of attending an initial assessment centre and refers to the age of the participant on the day they attended an Initial Assessment Centre, truncated to whole year.

10 principal components of population structure

The most widely used method to correct bias due to population stratification (PS) is principal components (PCs) analysis (PCA), but there is no objective method to guide which PCs to include as covariates. Often, the ten PCs with the highest eigenvalues are included to adjust for PS).

Definitions of criterion for frequency of alcohol drinking

There are several phenotype options that measure drinking behavior in the UKB (Data-Field 20414). After considering only phenotypes that cover the entire UKB sample, we were left with several to define alcohol per week. We used the sum of all alcoholic beverages per week as the weekly beverage phenotype for these respondents. Respondents who reported drinking less than once a week (one to three times a month,

or only on special occasions) were asked how many different types of alcoholic beverages they consumed per month. For these subjects, we added up the total amount of alcohol consumed each month and divided it by 4 to get an approximate weekly amount of alcohol consumed. Those who never drank had a code of 0.

Definitions of criterion for frequency of smoking

We selected several potential measures of smoking behavior in the UKB. These include: 1) ever-tobacco smoker status; 2) former tobacco smoker status (among ever-tobacco smokers); 3) number of cigarettes per day, based on the previous study. We coded ever smoker status as 1 if a respondent reported that they were a current or previous smoker, 0 if they reported never smoking and 0.5 if they reported only smoking less than once or twice per day. We coded cigarettes per day as 0 if ever-smoking status was also 0, otherwise, we used the maximum number of reported past or current cigarettes (or pipes/cigars) consumed per day.

Townsend deprivation index

We selected the index in the UKB (Data-Field 22189) Townsend deprivation index is a score representing the socioeconomic status of the individual based on the national census areas). They calculated immediately prior to participant joining UK Biobank. Based on the preceding national census output areas. Each participant is assigned a score corresponding to the output area in which their postcode is located.

The values here are rounded to 2 decimal places, replacing an earlier unrounded version.

Supplementary Table 1. Number of SNPs included for each of the proteins

Tissue	Proteins	Number of SNPs
Brain	Alcohol dehydrogenase [NADP(+)]	2
	Alpha-L-iduronidase	2
	Apolipoprotein E (isoform E2)	2
	Chitinase-3-like protein 1	4
	Complement C4b	3
	Copine-1	2
	Endoplasmic reticulum aminopeptidase 1	10
	Glutathione S-transferase P	2
	macrophage-stimulating protein	8
	Leukocyte immunoglobulin-like receptor subfamily B member 1	2
	Low affinity immunoglobulin gamma Fc region receptor II-a	2
	Low molecular weight phosphotyrosine protein phosphatase	6
	N-acylethanolamine-hydrolyzing acid amidase	4
	Serum amyloid A-1 protein	2
	Thioredoxin domain-containing protein 12	2
	Tumor necrosis factor receptor superfamily member 1B	2
	Tumor necrosis factor receptor superfamily member 6	2
CSF	6-phosphogluconate dehydrogenase, decarboxylating	2
	Adhesion G protein-coupled receptor E2	6
	ADP-ribosyl cyclase/cyclic ADP-ribose hydrolase 2	10
	Agouti-related protein	2
	Alcohol dehydrogenase [NADP(+)]	3
	Alpha-(1,3)-fucosyltransferase 5	3
	Alpha-L-iduronidase	7
	Arylsulfatase A	2
	B-cell lymphoma 6 protein	3
	C-C motif chemokine 16	2
	C-C motif chemokine 4-like	3
	C-type lectin domain family 7 member A	4
	Carbonic anhydrase 4	4
	Cathepsin B	3
	Cathepsin H	2
	Cathepsin S	4
	Cation-independent mannose-6-phosphate receptor	3
	Cell adhesion molecule-related/down-regulated by oncogenes	3
	Cerebral dopamine neurotrophic factor	5
	Chitinase-3-like protein 1	6
	Chitotriosidase-1	2
	CMRF35-like molecule 6	4

Coagulation factor VII	2
Complement C4b	46
Complement factor H-related protein 5	2
Contactin-2	2
Cystatin-M	7
Cysteine-rich with EGF-like domain protein 1	2
Cytoskeleton-associated protein 2	2
Dermatopontin	6
E-selectin	2
Endoplasmic reticulum aminopeptidase 1	17
Endothelial monocyte-activating polypeptide 2	2
Endothelin-converting enzyme 1	3
Fibroblast growth factor 19	3
Follicle stimulating hormone	2
Galectin-3	5
Gelsolin	2
Glutamate carboxypeptidase 2	25
Glutathione S-transferase P	7
Glypican-5	2
Granulins	2
Granulysin	4
Granzyme A	2
Growth hormone receptor	3
Haptoglobin	9
HemK methyltransferase family member 2	3
Hemopexin	10
Heparin cofactor 2	3
Hepatitis A virus cellular receptor 2	2
macrophage-stimulating protein	20
Hepatocyte growth factor activator	2
Hyaluronan and proteoglycan link protein 1	7
ICOS ligand	3
Intercellular adhesion molecule 1	3
Interleukin-1 receptor-like 1	17
Interleukin-1 Receptor accessory protein	3
Interleukin-1 receptor type 1	2
Interleukin-18 receptor 1	20
Interleukin-22	6
Interleukin-34	8
Interleukin-36 alpha	69

Interleukin-6 receptor subunit alpha	5
Interleukin-9	5
Kallikrein-8	2
Keratin, type I cytoskeletal 18	5
L-Selectin	10
Layilin	4
Leptin receptor	5
Leukocyte immunoglobulin-like receptor subfamily B member 1	3
Low affinity immunoglobulin gamma Fc region receptor II-b	7
Low molecular weight phosphotyrosine protein phosphatase	6
Luteinizing hormone	2
Lysozyme C	3
Membrane frizzled-related protein	47
Membrane metallo-endopeptidase-like 1	5
Vitamin K-dependent protein C	3
MHC class I polypeptide-related sequence B	80
Myeloid cell surface antigen CD33	4
N-acylethanolamine-hydrolyzing acid amidase	11
N-terminal pro-BNP	3
Netrin-4	3
Neutrophil collagenase	4
Nidogen-2	8
NKG2D ligand 3	3
Periostin	3
Plasma protease C1 inhibitor	3
Platelet-activating factor acetylhydrolase	10
Platelet-derived growth factor receptor beta	6
Platelet glycoprotein VI	2
Programmed cell death 1 ligand 1	4
Proprotein convertase subtilisin/kexin type 7	6
Proteasome activator complex subunit 1	4
Protein FAM3B	5
Protein FAM3D	5
Quinone oxidoreductase-like protein 1	3
Scavenger receptor class F member 1	2
Secreted frizzled-related protein 3	2
Semaphorin-3E	6
Sialic acid-binding Ig-like lectin 14	3
Sialic acid-binding Ig-like lectin 9	2
SLAM family member 5	7

	SLAM family member 7	5
	Small nuclear ribonucleoprotein F	7
	Tenascin	13
	Teratocarcinoma-derived growth factor 1	12
	Thioredoxin domain-containing protein 12	4
	Thrombospondin-4	2
	Tissue factor pathway inhibitor	2
	Transmembrane glycoprotein NMB	3
	Tryptase beta-2	5
	Tumor necrosis factor-inducible gene 6 protein	2
	Tumor necrosis factor ligand superfamily member 15	2
	Tumor necrosis factor receptor superfamily member 6	3
	Vitronectin	8
	WAP, Kazal, immunoglobulin, Kunitz and NTR domain-containing protein 2	4
**Plasma**	ADP-ribosyl cyclase/cyclic ADP-ribose hydrolase 2	6
	Alcohol dehydrogenase [NADP(+)]	2
	Alpha-L-iduronidase	3
	Angiogenin	2
	Apolipoprotein L1	4
	Beta-endorphin	4
	Calcium/calmodulin-dependent protein kinase type 1	3
	Cathepsin S	3
	Cation-independent mannose-6-phosphate receptor	2
	CD209 antigen	3
	CD27 antigen	2
	Chitinase-3-like protein 1	5
	Chitotriosidase-1	2
	Coagulation factor VII	3
	Coagulation Factor XI	4
	Complement factor H-related protein 5	2
	Cystatin-F	2
	Cystatin-SA	3
	Cysteine-rich with EGF-like domain protein 1	3
	Dual specificity mitogen-activated protein kinase kinase 4	3
	E-selectin	5
	Ectonucleoside triphosphate diphosphohydrolase 5	2
	Endoplasmic reticulum aminopeptidase 1	5
	Ficolin-2	2
	Galectin-3	3

Granulins	2
Haptoglobin	20
Hemopexin	8
Hepatitis A virus cellular receptor 2	2
Histidine-rich glycoprotein	4
Intercellular adhesion molecule 1	4
Interleukin-1 receptor-like 1	5
Interleukin-1 Receptor accessory protein	3
Interleukin-17 receptor A	2
Interleukin-6 receptor subunit alpha	3
Leukocyte immunoglobulin-like receptor subfamily B member 1	3
Low molecular weight phosphotyrosine protein phosphatase	2
Lysozyme C	2
Melanoma-derived growth regulatory protein	3
MHC class I polypeptide-related sequence A	51
Myeloid cell surface antigen CD33	2
N-acylethanolamine-hydrolyzing acid amidase	3
Non-histone chromosomal protein HMG-14	2
Phospholipase A2, membrane associated	4
Plasma protease C1 inhibitor	2
Platelet-derived growth factor receptor alpha	3
Platelet-derived growth factor receptor beta	4
Platelet glycoprotein VI	2
Protein FAM3D	3
Protein Z-dependent protease inhibitor	2
Scavenger receptor class F member 1	3
Semaphorin-3E	5
Sialic acid-binding Ig-like lectin 6	4
Tenascin	3
Teratocarcinoma-derived growth factor 1	7
Thioredoxin domain-containing protein 12	16
Ubiquitin carboxyl-terminal hydrolase 25	8
Vascular endothelial growth factor receptor 3	2
Vitamin K-dependent protein C	4

Supplementary Table 2 The result of MR analysis of MSP (exposure) and fluid intelligence score (outcome)

Analysis method	MSP (brain)→ fluid intelligence score		MSP(CSF)→ fluid intelligence score	
	Estimate (Estimate 95%CI/SE/df)	*P-value*	Estimate (Estimate 95%CI/SE/df)	*P-value*
Inverse variance weighted	0.42（0.41，0.43）	$< 5.00\times10^{-8}$	0.31（0.30，0.33）	$< 5.00\times10^{-8}$
Weighted median	0.44（0.43，0.46）	$< 5.00\times10^{-8}$	0.25（0.25，0.26）	$< 5.00\times10^{-8}$
Simple mode	0.46（0.42，0.50）	1.96×10^{-71}	0.24（0.22，0.25）	——
Weighted mode	0.45（0.42，0.48）	2.11×10^{-89}	0.25（0.24，0.25）	——
MR-Egger	0.54（0.49，0.60）	1.64×10^{-61}	0.09（0.06，0.11）	1.60×10^{-12}
Horizontal pleiotropy				
Egger-intercept	-1.57×10^{-2}（3.08×10^{-3}）	6.65×10^{-7}	3.57×10^{-2}（1.70×10^{-3}）	2.95×10^{-75}
Heterogeneity				
MR-Egger	290.18 (302)	6.77×10^{-1}	3432.20 (669)	$< 5.00\times10^{-8}$
Inverse variance weighted	315.98 (303)	2.92×10^{-1}	5681.52 (670)	$< 5.00\times10^{-8}$

Abbreviation: MR, Mendelian randomization, MSP: macrophage-stimulating protein, IQ: Intelligence Quotient, CSF: cerebrospinal fluid, SE: standard error, df: degree of freedom.

Estimates of the β (95%CI) P-value from MR-Egger, estimates of the Cochran Q statistics (df) P-value from Cochran Q test and estimates of the Intercept (SE) P-value from MR-Egger-intercept.

P < 0.05/2 indicates statistical significance.
